# Maladies Produced by Boots and Shoes

**Published:** 1874-10

**Authors:** James Paget


MALADIES PRODUCED BY BOOTS AND SHOES, AND THEIR TREATMENT. *By Sir James Paget.*—Maladies depending on the wearing of too small and badly fitting boots are many and varied, such as deformaties of the toes, bunions, corns, in-growing nails, painful bursæ, etc. In order to study deformaties of the toes, we should first obtain a good idea of a perfect foot. In a perfect female foot we find: 1. Great width and fullness of instep. 2. Well marked great toe. 3. Long second toe, projecting a little beyond great toe. 4. Very small, or in some cases almost suppressed, little toe.

In the old classic statues the second toe always projects beyond the first, but that natural type of foot is now rarely seen. The modern great toe generally extends beyond the second, invariably so in people with flat feet.

In the male the great toe is not quite so prominent as the second. The feet of all persons cannot be deformed, nor can corns and bunions be produced in every one. It is doubtless owing to their complete reactive nutrition, the repair that takes place in the night being more than enough for the day's waste. This is not impossible, when we remember the complete repair that occurs after great muscular waste, as in athletics. The diseases caused by the pressure and friction of boots are thus classified:

I. *Mutual Compression of the Toes.* Naturally there is a considerable interval between the first and second toes, and a less degree between the others, so that when the foot bears the weight of the body each toe is free from contact with its fellow; hence in wet clay you would receive a separate impression of each. In the deformity, though, which is produced by small boots, the toes are squeezed together so as to form a transverse arch, the first and second toes then only bearing the weight of the body. Thus there are formed—

1. Soft corns between the toes by their friction on each other.

2. Hard corns on outer side of little toe and inner side of great toe, and projecting points pressed upon.

3. Complete immobility of the toes, except the great one. The natural mobility in civilized nations does not exist now in more than about one person in five hundred.

4. Painful bursæ between metatarsal bones.

5. In extreme cases corns and chafed spots are produced by the squeezing and rubbing together of the pads of the great and and little toes. Kid gloves, though worn continually, never cause bunions, since the kid stretches to the hands; but in the manufacture of boots, especially ladies' boots, unyielding canvas is used to line them, so that the leather is prevented from stretching and showing the true shape and size of the foot. The foot enlarges when bearing the weight of the body, and also toward evening; hence a boot thus made from a measure taken when the foot is suspended in the air, and in the morning, is too small for the foot in the evening. Women's feet are generally measured in the air, but men's when they are standing on them. The high heels in ladies' boots, too, cause them to be always walking down hill, however level the path may be, thus driving the foot more and more to the front.

II. *Deflection of the Toes* falls chiefly on the great toe, the result of wearing—

1. Boots too narrow in front.

2. Boots (now out of fashion) having the point in a line with the centre of the head; the great toe, which naturally is in a line with the inner side of the heel, being deflected outward toward the point.

3. Short boots especially. In them the great toe is brought sharply in contact with the end, and as the tarsus and metatarsus will not yield much, and the metatarso-phalangeal joint will, a deflection of the great toe takes place outward, and sometimes downward. This is the most frequent and worst form. This deflection of the great toe is the source of great trouble, as bunions occur over the metatarso-phalangeal joint, soft corns on the second, third and fourth toes, under which it lies, and worst of all, a total loss of movement in the great toe.

*Treatment of the above Deformities.* If just beginning, keep the toes apart by pads of plaster. Isinglass plaster upon felt is the best. The pad must be worn day and night. Of course bad boots must be left off. The treatment by night is even more important than that during the day; for then especially repair goes on, and the least relaxation in the night more than undoes the good done in the day. Sometimes it has been considered necessary to divide the tendons, but these do not produce the deformity; they merely adapt themselves to it. If they are divided, the deep-seated fibrous textures should be divided as well. In the worst cases the great toe has to be amputated.

There are also certain deformities of the second toe, but it is doubtful whether these deformities are due to the wearing of bad boots, as sometimes they are hereditary. The third and fourth toes have no special deformities; they suffer only by being lifted up or pushed down. The little toe sometimes almost disappears, from atrophy caused by pressure.—*Herald of Health.*

